# Podocan Promotes Differentiation of Bovine Skeletal Muscle Satellite Cells by Regulating the Wnt4-β-Catenin Signaling Pathway

**DOI:** 10.3389/fphys.2019.01010

**Published:** 2019-08-07

**Authors:** Shuang Li, Dan Liu, Yuying Fu, Chunyu Zhang, Huili Tong, Shufeng Li, Yunqin Yan

**Affiliations:** The Laboratory of Cell and Development, Northeast Agricultural University, Harbin, China

**Keywords:** podocan, differentiation, bovine, skeletal muscle satellite cells, Wnt4/β-catenin

## Abstract

**Background:**

Small leucine-rich repeat proteins (SLRPs) are highly effective and selective modulators of cell proliferation and differentiation. Podocan is a newly discovered member of the SLRP family. Its potential roles in the differentiation of bovine muscle-derived satellite cells (MDSCs) and its underlying functional mechanism remain unclear. Our study aimed to characterize the function of the podocan gene in the differentiation of bovine MDSCs and to clarify the molecular mechanism by which podocan functions in order to contribute to a better understanding of the molecular mechanism by which extracellular matrix promotes bovine MDSC differentiation and provide a theoretical basis for the improvement of beef quality.

**Methods:**

Bovine MDSCs were transfected with vectors to overexpress or inhibit podocan, and podocan protein was added to differentiation culture medium. qRT-PCR, western blotting, and immunofluorescence were performed to investigate the effects of podocan on MDSC differentiation. Confocal microscopy and western blotting were used to assess the nuclear translocation and expression of β-catenin. An inhibitor and activator of β-catenin were used to assess the effects of the Wnt/β-catenin signaling pathway on MDSC differentiation. We inhibited β-catenin while overexpressing podocan in MDSCs. Then, we performed mass spectrometry to identify which proteins interact with podocan to regulate the Wnt/β-catenin signaling pathway. Finally, we confirmed the relationship between podocan and Wnt4 by co-immunoprecipitation and western blotting.

**Results:**

Podocan protein expression increased significantly during bovine MDSC differentiation. Differentiation of bovine MDSC was promoted and suppressed by podocan overexpression or inhibition, respectively. Podocan was also shown to modulate the Wnt/β-catenin signaling pathway. Treatment of bovine MDSCs with β-catenin inhibitor and activator showed that the Wnt/β-catenin pathway is involved in bovine MDSC differentiation. Furthermore, the effect of podocan on bovine MDSC differentiation was suppressed when this pathway was inhibited. We also found that podocan interacts with Wnt4. When Wnt4 was inhibited, podocan-induced promotion of bovine MDSC differentiation was attenuated through Wnt/β-catenin signaling.

**Conclusion:**

Podocan regulates Wnt/β-catenin through Wnt4 to promote bovine MDSC differentiation.

## Introduction

The growth, development, and regeneration of adult skeletal muscles in animals depend on muscle-derived satellite cells (MDSCs). Bovine MDSCs can be further differentiated to form myotubes and muscle fibers through cell fusion. Therefore, the myotube fusion rate is an important indicator of the degree of muscle cell differentiation (Rudnicki et al., 2008; Wang and Rudnicki, 2011). During the development of vertebrate embryos, mononuclear proliferative myoblasts exit the cell cycle to increase in number and then differentiate and fuse to become multinuclear myotubes (Sabourin and Rudnicki, 2000; Chargé and Rudnicki, 2004). Skeletal muscle growth is regulated by multiple factors, among which MyoG and MYH3 are markers of muscle differentiation in cattle (Wagers and Conboy, 2005; Singh et al., 2015). Moreover, skeletal muscle growth is affected by multiple signaling pathways, such as mTOR, Notch, TGF-β, and Wnt (Conboy et al., 2003; Winbanks et al., 2012). Among the signaling proteins, the expression of members of the Wnt family is known to be important for skeletal muscle development and regeneration (Tajbakhsh et al., 1998; Brack et al., 2007; Nemeth et al., 2007; Park et al., 2007). Mechanistically, Wnt/β-catenin signaling plays a vital role in the differentiation of bovine MDSCs. The Wnt family can be divided into two categories, namely the canonical and non-canonical pathways (Church and Francis-West, 2002; Veeman et al., 2003). The Wnt/β-catenin cascade represents the classical Wnt pathway and plays an important role in various biological activities in stem cells (Zhang et al., 2018). Canonical Wnt/β-catenin signaling acts via the transcriptional co-activator β-catenin and is known to be critical for skeletal muscle myogenesis during embryonic development, however, its role in bovine MDSCs remains unknown (Stern et al., 1995; Ikeya and Takada, 1998; Agley et al., 2017).

Recently, it was discovered that small leucine-rich repeat proteins (SLRPs) in the extracellular matrix (ECM) play important roles in regulating the proliferation and differentiation of specific cells. For example, fibromodulin (fibrinomodulin) regulates the differentiation of myoblasts to form myocytes (Lee et al., 2018), while biglycan and decorin play important roles in tendon injury repair (Dunkman et al., 2014). Further, biglycan promotes the proliferation and migration of vascular smooth muscle cells (Shimizu-Hirota et al., 2004a). These observations indicate that members of the SLRP protein family can influence muscle development.

Regarding the molecular mechanism of action of these proteins, it is generally believed that SLRP family proteins form a regulatory network outside of the cell and mediate a variety of signaling cascades, thereby affecting cell behavior (Schaefer and Iozzo, 2008; Nastase et al., 2014). As extracellular glycoproteins, most members of the SLRP protein family covalently bind glycosaminoglycans to form proteoglycans, which activate various cell surface receptors, growth factors, cytokines, and other ECM components to influence cell function and activate the Wnt/β-catenin and TGF-β signaling pathways (Dellett et al., 2012). We recently reported that that podocan can affect the Wnt pathway in C2C12 cells, but it is unknown whether it also plays the same role in bovine muscle satellite cells. More importantly, that finding did not explore how podocan as an extracellular matrix protein regulates the Wnt signaling pathway (Liu et al., 2019). This is very important for meat quality improvement and breeding.

Podocan belongs to the fifth class of the SLR protein family and is expressed in the sclerotic glomerular lesions of experimental human immunodeficiency virus-associated nephropathy (HIVAN) (Ross et al., 2003; Shimizu-Hirota et al., 2004b). Podocan is involved in kidney function and may represent a unique therapeutic target for the treatment of diabetic nephropathy (Nio et al., 2017). Moreover, a lack of podocan results in excessive arterial repair and prolonged smooth muscle cell (SMC) proliferation, which is likely mediated by the Wnt/β-catenin pathway (Hutter et al., 2013). However, the molecular association between podocan and the classical Wnt/β-catenin signaling pathway is unclear. Furthermore, there are few studies on the function and mechanism of action of podocan.

In this study, we aimed to determine the effect of podocan on MDSC differentiation *in vitro* and to investigate the relationship between podocan and the Wnt/β-catenin pathway. This study provides new theoretical and experimental data for the study of bovine skeletal MDSC differentiation.

## Materials and Methods

### Cell Isolation, Culture, and Differentiation

All experiments involving animals conformed to internationally accepted standards and were approved by the Animal Welfare Committee of Northeast Agricultural University, Harbin, China. The isolation of bovine skeletal MDSCs was performed as follows. Skeletal muscle tissues were pooled and finely minced. They were then treated with 0.2% collagenase I (Sigma-Aldrich, St. Louis, MO, United States) for 2 h, followed by treatment with 0.25% trypsin (Sigma-Aldrich) for 30 min. The digested tissues were filtered through a 400 mesh, which was rinsed with phosphate-buffered saline (PBS). The cells were collected by centrifugation at 800 × *g* and washed once with PBS. Aliquots were added to cell culture plates coated with polylysine (Sigma-Aldrich). Subsequently, the isolated bovine MDSCs were cultured in Dulbecco’s modified Eagle’s medium containing 20% fetal bovine serum (FBS), 10% horse serum, 100 U/ml penicillin, and 100 μg/ml streptomycin at 37°C in 5% CO_2_ and 95% air. When cells reached 50–60% confluence, they were washed once with PBS, passaged in cell culture flasks, and seeded into 6-well plates. After subsequent culture to 70–80% confluence, the medium was discarded, and cells were cultivated in differentiation medium (DM) composed of 2% horse serum (Gibco, Grand Island, NY, United States). Bovine MDSCs were allowed to differentiate for various amounts of time *in vitro*.

### Construction of Vectors

#### Construction of Podocan Overexpression Vector

Full-length podocan cDNA was amplified by PCR from MDSC total cDNA using the following primers: 5′-AAGCTTATGGAAGGAGCCCACGC-3′ and 5′-GAATTCCT ATCGCCTTTCTTCTTCCTCC-3′. In-frame ligation of the podocan cDNA into the pCMV-N-His vector (Beyotime Biotechnology, Beijing, China) was performed according to the manufacturer’s instructions, and the construct was sequenced by Sangon Biotech China to ensure fidelity.

#### Construction of CRISPR/dCas9 Inhibition Vectors Targeting Podocan and Wnt4

Three sgRNAs targeting different sites in the podocan (NCBI Gene ID: 509606) promoter (from −522 to + 101) and four sgRNAs targeting sites in the *Wnt4* (NCBI Gene ID: 789600) promoter (from + 15103 to + 16486) were designed and are shown in Table 1. These oligonucleotides were cloned into the pSPgRNA expression vector (Addgene, Cambridge, MA, United States) after synthesis by Sangon Biotech. The obtained sgRNA vectors for podocan and *Wnt4* were transfected with dCas9 into bovine MDSCs, which were cultured for 48 and 72 h in 6-well plates. Each well was then co-transfected with 2 μg dCas9 and an equal amount of the sgRNA expression plasmids with Lipofectamine 2000 (Invitrogen, Carlsbad, CA, United States). After transfection for 48 and 72 h, total RNA and protein were collected using TRIzol reagent (Invitrogen) and RIPA buffer (Beyotime Biotechnology), respectively, and stored at −80°C. Because the CRISPR/dCas9 system targets the promoters, there may be off-target effects. We therefore screened for the most efficient vectors for the inhibition of podocan and Wnt4 using western blotting.

**TABLE 1:** Sequences of primers targeting podocan and Wnt4.

**Gene**	**Single-guide (sgRNA) sequence**
Podocan1 (P1)	CACC GTGCCTGTGTCCGGAGG
Podocan2 (P2)	CACC GGGGTCGGGGCGTGGTACGG
Podocan3 (P3)	CACC GCACGCGGGGGGGTGGGGGC
Wnt1 (W1)	CACC GGCTTGGAGTGGACAAGGTC
Wnt2 (W2)	CACC GTCACGGGGGTCCCTGCCTG
Wnt3 (W3)	CACC GAGTGCCACCGCTGTGATTC
Wnt4 (W4)	CACC GCTCTGTCCTCCGCTGAAAT

### Purification of Proteins

The 293T cells were transfected with pCMV-N-His-podocan, and the medium was collected. Proteins were purified using the pET system according to the manufacturer’s instructions.

### qRT-PCR and Western Blotting

#### qRT-PCR

Total RNA was extracted from bovine MDSCs after transfection and subsequent differentiation for 48 and 72 h. cDNA was obtained by reverse transcription (TransGen Biotech, Beijing, China) using total RNA according to the manufacturer’s instructions. qRT-PCR analysis was used to detect podocan, *MYH3*, and *MyoG* mRNA expression, and β-actin was used as an internal reference gene. The primer sequences are listed in Table 2.

**TABLE 2:** Sequences of primers used for quantitative RT-PCR.

**Gene**	**Forward primer (5′–3′)**	**Reverse primer (5′–3′)**
Podocan	GACGCTGAACCTCCAGAACAA	GACCAAAGGTGAGCCCGTAGA
MYH3	TGCCAAGGGGAGAATCAA	TCCAGGAGGTGTAGCGGT
MyoG	GACTCAAGAAGGTGAATGAAGCC	TATTATAGTGCGCTGCCCCAC
β-actin	GACCTCTACGCCAACACG	GCAGCTAACAGTCCGCCTA

#### Western Blotting

To assess podocan, MYH3, MyoG, and Wnt4 expression, we collected proteins from bovine MDSCs that were transfected with vectors and treated with small molecules using ice-cold RIPA buffer (Beyotime Biotechnology). Proteins were then separated by 10% SDS-PAGE, transferred to PVDF membranes (Millipore Watford, United Kingdom), and used for western blotting. Membranes were probed using anti-podocan (GeneTex, Irvine, CA, United States), anti-MYH3 (Proteintech, Wuhan, China), anti-MyoG (Santa Cruz Biotechnology, Santa Cruz, CA, United States), anti-β-catenin (Abcam, Cambridge, MA, United States), anti-phospho-β-catenin (S33) (Abcam), and anti-Wnt4 (Bioss Antibodies, Beijing, China) antibodies overnight at 4°C. The next day, the membranes were washed four times with PBS containing 5% Tween-20 (PBST) for 8 min each. They were then incubated with goat anti-rabbit-HRP (Bioss Antibodies, Beijing, China) secondary antibody at 37°C for 1 h and washed with PBST four times for 8 min each. The Super ECL Plus kit (Applygen Technologies Inc., Beijing, China) was used to visualize protein bands with a small chemiluminescence imager (Sage Creations, Beijing, China) for imaging.

### Immunofluorescence and Myotube Analysis

To detect changes in cell morphology, cells were fixed with cold methanol for 20 min after transfection. For permeabilization, cells were washed with PBS containing 0.5% TritonX-100 (PBST) and incubated for 2 h in PBS containing 5% BSA and 0.5% TritonX-100 at 37°C. After blocking, the cells were incubated for 2 h with the primary antibodies anti-podocan (diluted 1:50), anti-MYH3 (diluted 1:100), anti-β-catenin (diluted 1:100), or anti-desmin (Santa Cruz Biotechnology, diluted 1:50), which were diluted in PBST containing 5% BSA. Next, cells were washed with PBST four times. The samples were incubated with fluorescently labeled rabbit IgG or RBITC-labeled rabbit IgG secondary antibody (Biosynthesis Biotechnology, China, diluted 1:20) in the dark at 37°C for 2 h, and cells were then washed with PBST four times. DNA was stained for 3 min with DAPI. All images were taken using a fluorescence microscope (Olympus, Tokyo, Japan).

The fusion index was determined by calculating the percentage of nuclei in myotubes relative to the total number of nuclei. Each image contained more than 500 nuclei, and five fields of view were assessed for each experimental condition. The myotube index was analyzed using ImageJ software (National Institutes of Health, Bethesda, MD, United States).

### Activator and Inhibitor Treatment

LiCl (Sigma-Aldrich) was used as an activator of the Wnt signaling pathway. In this study, cells were treated with a concentration of 10 mM for 48 h after differentiation. The same concentration of NaCl (Sigma-Aldrich) was used as a control. XAV-939 (Selleck Chemicals, Houston, TX, United States), an inhibitor of β-catenin, was used at 1 μM for 48 h after differentiation, and an equivalent amount of DMSO was used as the control.

### Co-immunoprecipitation

Bovine MDSCs were lyzed in 1 ml of RIPA buffer after differentiation. Then, 20 μl of protein extract was used as an input. The remaining portion was divided equally into two equal samples, to which 2 μg of rabbit IgG (Beyotime Biotechnology Beijing, China) and indicated antibodies were added at 4°C overnight. Subsequently, protein A + G (Beyotime Biotechnology) was incubated with the samples at 4°C for 3 h. After centrifugation, the beads were washed five times with RIPA buffer, and the harvested beads were resuspended in the same volume of 5 × SDS sample buffer and boiled for 10 min to remove the Sepharose beads. Then, the immunoprecipitates were analyzed by western blotting. Next, the gel was soaked in Coomassie Brilliant Blue for 30 min, and the color was eluted overnight by acetic acid. The entire gel was sent to Applied Protein Technology (Shanghai, China) for mass spectrometry-based sequencing. Based on the sequencing results, a protein that may bind to podocan and exert biological functions was selected. The above procedure was carried out using a specific antibody against the selected protein, and a co-immunoprecipitation assay was used to precipitate the podocan protein.

### Statistical Analysis

All results are expressed as mean ± SEM. Analysis of statistical significance between two groups was performed using the Student’s *t*-test for two group comparisons or one-way ANOVA was used for multiple group comparisons. Differences were regarded as significant at ^*^p < 0.05, whereas ^∗∗^*p* < 0.05. All statistical tests were performed using Prism software (GraphPad Software Inc., La Jolla, CA, United States) and SPSS 12.0 (SPSS Inc., Chicago, IL, United States).

## Results

### Expression of Podocan in Bovine MDSCs at Various Stages of Differentiation

**FIGURE 1:**
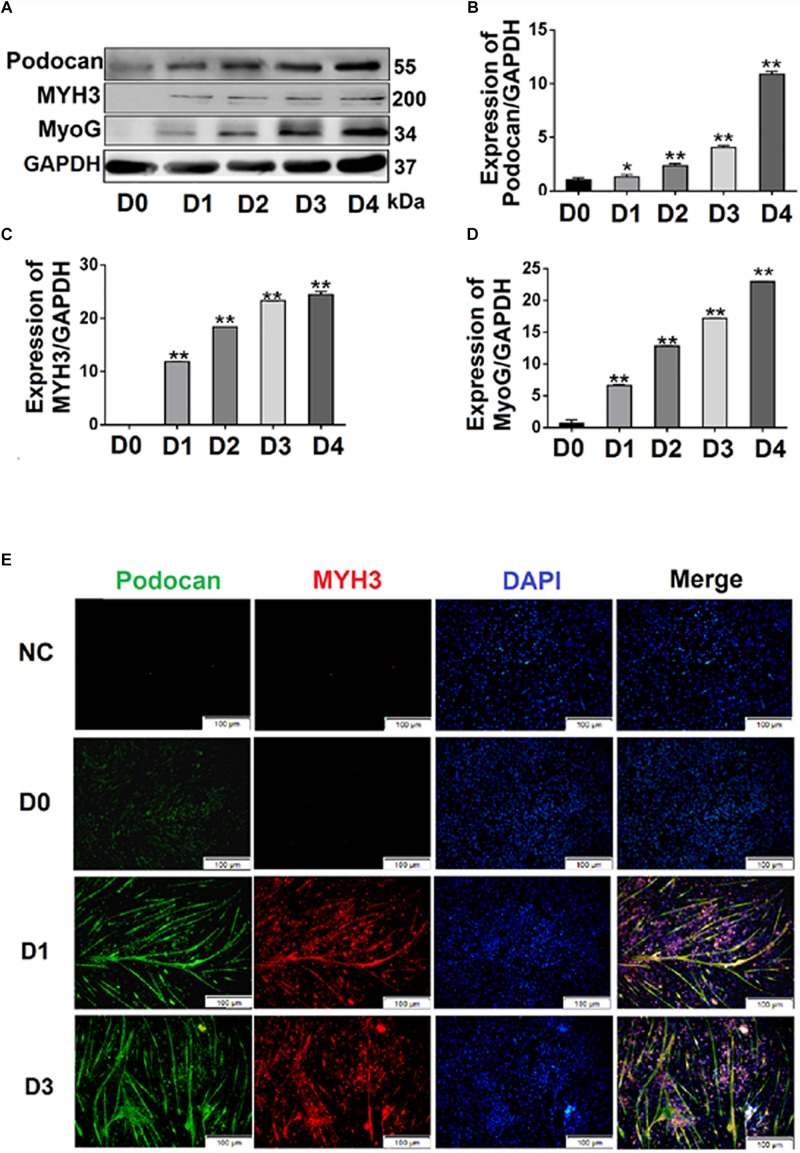
Expression of podocan during the differentiation of bovine muscle-derived satellite cells (MDSCs). **(A)** Bovine MDSCs were induced to differentiate for different durations. D0, MDSCs cultured in proliferation medium. D1, D2, D3, and D4, MDSCs cultured in differentiation medium for 1, 2, 3, or 4 days, respectively. Western blotting was performed to examine podocan, MYH3, and MyoG protein expression. **(B–D)** Quantification of podocan, MYH3 and MyoG protein expression in panel **(A)**. The results detected that podocan, MYH3 and MyoG expression were all increased significantly, compared with the proteins expression in D0. **(E)** Immunofluorescence staining for podocan (green), MYH3 (red), and total nuclei (blue) after MDSC differentiation at different stages. D0, D1, D3, indicate cells induced to differentiate for 0, 1, or 3 days, respectively (*n* = 3). NC, negative control. Scale bar: 100 μm. Statistical significance was calculated using the *t*-test; ^*^p < 0.05, ^∗∗^*p* < 0.01; ns, no significant difference.

We cultured bovine MDSCs in proliferation medium to 60–70% confluence (day 0, D0), then replaced the medium with DM. MDSCs fused and formed short myotubes (D1). The next few days of differentiation were sampled as D2, D3, and D4. Single cells were fused into multinucleated myotubes, and phase contrast microscopy images of morphological changes are shown in Supplementary Figure S1. Western blotting was performed to detect the protein expression of podocan during each stage of bovine MDSC differentiation *in vitro*. Western blotting raw dates showed in Supplementary Figure S4. The results demonstrated that expression levels in samples from D2 to D4 were significantly higher than those from D0 (Figures 1A,B). Statistical analysis also showed that the expression of podocan gradually increased with an increasing degree of cell differentiation. Moreover, when compared to those on D0, the protein levels of the muscle cell differentiation-related factors MYH3 and MyoG were significantly higher on subsequent days (*p* < 0.01; Figures 1A,C,D).

Immunofluorescence analyses of bovine MDSCs were performed on D0, D1, and D3. Before bovine MDSC differentiation, myotubes were not observed, and the intensities of the podocan and MYH3 fluorescent signals were weak. After differentiation was induced, however, the MDSCs fused and formed long myotubes. The expression levels of podocan and MYH3 gradually increased throughout cell differentiation (Figure 1E).

### Effect of Podocan on Bovine MDSC Differentiation

#### Overexpression of Podocan Promotes Bovine MDSC Differentiation

**FIGURE 2:**
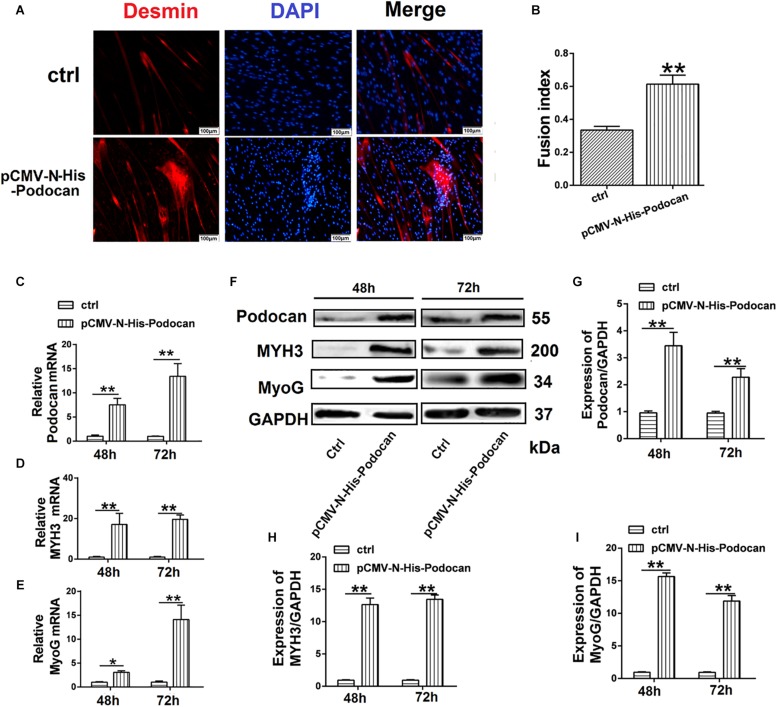
Effects of podocan overexpression on bovine muscle-derived satellite cell (MDSC) differentiation. **(A)** Immunofluorescence staining of desmin (Red) expression in bovine MDSCs at 48 h of differentiation with podocan overexpression induced by pCMV-N-His-Podocan transfection. pCMV-N-His empty vector was transfected for the control group; nuclei were labeled with DAPI and are shown in blue. Magnification 200×. **(B)** Quantification of bovine MDSC fusion index of data described in (A). The resulted showed that fusion index increased significantly after overexpressed podocan. **(C–E)** Podocan, *MYH3*, and *MyoG* mRNA expression during bovine MDSC differentiation for 48 or 72 h after podocan overexpression. β-actin was used as a reference gene for quantification (*n* = 3). QRT-PCR results showed that the MYH3 and MyoG mRNA were increased after podocan mRNA increased. **(F)** Podocan, MYH3, and MyoG protein expression during bovine MDSC differentiation for 48 and 72 h after podocan overexpression. GAPDH was used as a reference protein for analysis. **(G–I)** Quantification of podocan, MYH3, and MyoG protein expression from panel **(F)**. The protein expression levels of podocan, MYH3, and MyoG were higher in the overexpression podocan group than in the control (ctrl) group. Statistical significance was calculated using the *t*-test; ^*^p < 0.05, ^∗∗^*p* < 0.01. Scale bar: 100 μm.

To investigate the effect of podocan on MDSC differentiation, we used pCMV-N-His-Podocan to overexpress podocan in bovine MDSCs. The myotube fusion increased after overexpression of Podocan. The phase contrast microscopy images of morphological changes are shown in Supplementary Figure S2. Cells were fixed at 48 h for desmin immunofluorescence staining (Figure 2A). The myotube fusion index in the podocan overexpression group was significantly higher (27.66%) than that in the control group (Figure 2B). In addition, in the podocan overexpression group, the mRNA expression of podocan was significantly upregulated after bovine MDSC differentiation for 48 and 72 h, as compared to levels in the corresponding control group (Figure 2C). Moreover, *MYH3* and *MyoG* levels also increased significantly after podocan overexpression and differentiation for 48 and 72 h (Figures 2D,E). As expected, western blotting for podocan, MYH3, and MyoG showed similar expression patterns (Figures 2F–I and Supplementary Figure S5). In summary, these results demonstrate that podocan overexpression promotes bovine MDSC differentiation.

#### Podocan Suppression Inhibits Bovine MDSC Differentiation

**FIGURE 3:**
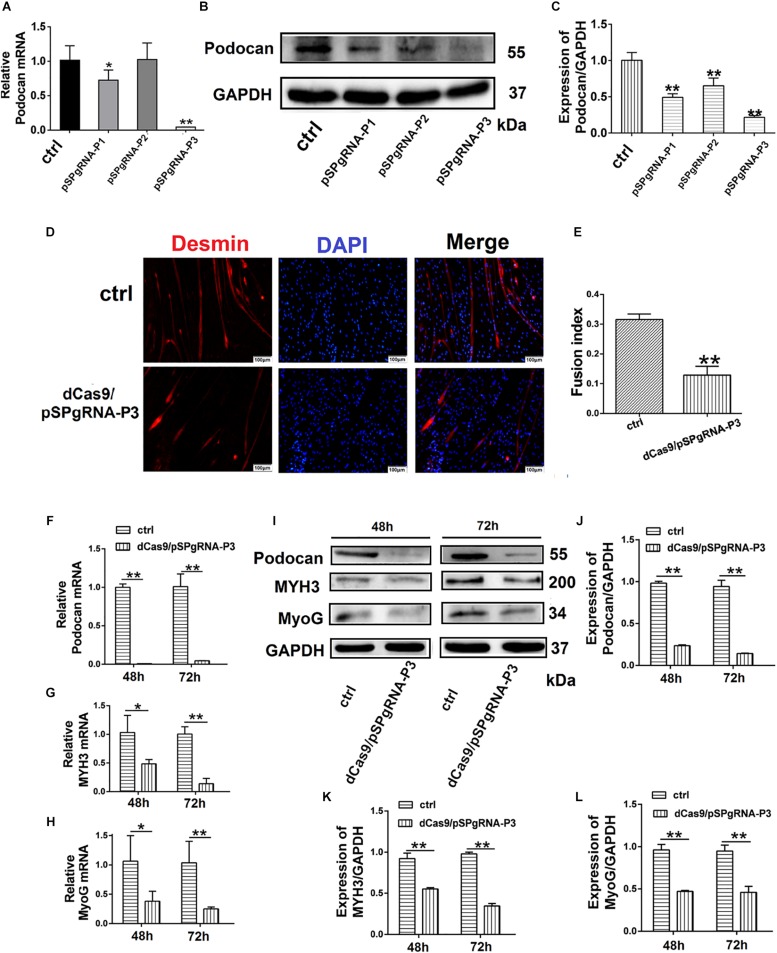
Suppression of podocan inhibits bovine muscle-derived satellite cell (MDSC) differentiation. **(A)** MDSCs differentiated for 48 h after co-transfection with dCas9 and pSPgRNA as control (Ctrl) or pSPgRNA-P(1–3). QRT-PCR was used to detect podocan mRNA expression. **(B)** Podocan protein expression after co-transfection with dCas9 and pSPgRNA-P(1–3) vectors. **(C)** Quantification of podocan protein expression from panel **(B)**. QRT-PCR and western blotting results showed that podocan protein expression was lowest in co-transfected with dCas9 and pSPgRNA-P3 group. **(D)** Immunofluorescence detection of desmin (Red) after co-transfection of dCas9 and pSPgRNA-P3 or pSPgRNA and differentiation for 48 h. pSPgRNA was used as a control. Cell nuclei are shown in blue. Scale bar: 100 μm. **(E)** Quantitative analysis of myotube fusion index of desmin-expressing cells as described in (D). Fusion index decreased significantly after inhibited podocan expression. **(F–H)** Podocan, *MYH3*, and *MyoG* mRNA expression after co-transfection of dCas9 and pSPgRNA-P3 after podocan inhibition in bovine MDSCs differentiated for 48 and 72 h (*n* = 3). QRT-PCR results showed that the MYH3 and MyoG mRNA were decreased after podocan mRNA decreased **(I)** Podocan, MYH3, and MyoG protein expression after podocan inhibition in bovine MDSCs differentiated for 48 and 72 h. **(J–L)** Quantification of podocan, MYH3, and MyoG protein expression from panel **(I)**. The protein expression levels of podocan, MYH3, and MyoG were lower in the inhibition podocan group than in the control (ctrl) group. Statistical significance was calculated using the *t*-test; ^*^p < 0.05, ^∗∗^*p* < 0.01.

To further determine the effect of podocan on the differentiation of bovine MDSCs, we used CRISPRi for transcriptional inhibition. We designed three plasmid vectors, namely pSPgRNA-P1, pSPgRNA-P2, and pSPgRNA-P3, which were separately co-transfected with the dCas9 plasmid vector into bovine MDSCs for 48 h; pSPgRNA was used for the control group. Podocan mRNA expression was reduced by 99.92% (*p* < 0.01) in the pSPgRNA-P3 group compared to its expression in the controls (Figure 3A). Moreover, western blotting showed that podocan protein expression was significantly reduced (*p* < 0.01), especially in MDSCs co-transfected with dCas9 and pSPgRNA-P3 compared to that in MDSCs co-transfected with dCas9 and pSPgRNA (Figures 3B,C). Therefore, pSPgRNA-P3 was selected for subsequent experiments. The myotube fusion decreased after knockdown expression of Podocan. The phase contrast microscopy images of morphological changes are shown in Supplementary Figure S3. After co-transfecting the pSPgRNA-P3 and dCas9 plasmid vectors for 48 h, changes in the myotube fusion index of bovine MDSCs during *in vitro* differentiation were determined by desmin staining to study the effects of podocan inhibition on differentiation (Figure 3D). As a result, upon podocan inhibition, the myotube fusion index was found to be reduced to 18.7% (*p* < 0.01) of the control level (Figure 3E).

Bovine MDSCs were then co-transfected with pSPgRNA or pSPgRNA-P3 and dCas9 and allowed to differentiate for 48 and 72 h. At 72 h after differentiation, there was a significant decrease in podocan mRNA expression (Figure 3F), which was accompanied by decreases in *MYH3* and *MyoG* mRNA expression (*p* < 0.01), as compared to control levels (Figures 3G,H). Moreover, based on western blotting raw dates in Supplementary Figure S6, the inhibition of podocan was accompanied by decreases in MYH3 and MyoG protein levels. All of these changes were statistically significant (*p* < 0.01) at each time point (Figures 3I–L). These results indicate that suppression of podocan expression inhibits bovine MDSC differentiation *in vitro*.

#### Podocan Added to DM Affected Bovine MDSC Differentiation

**FIGURE 4:**
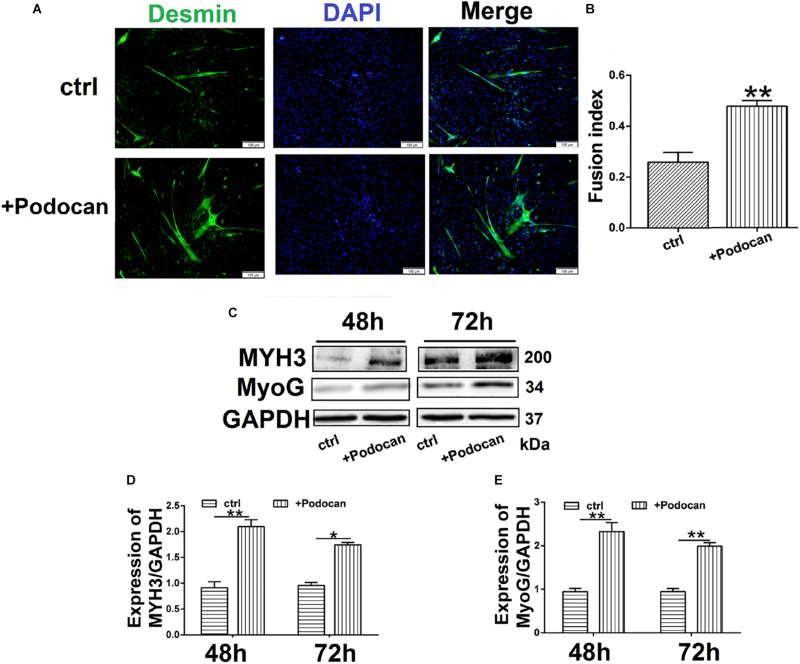
Podocan were added in DM affected bovine MDSC differentiation. Bovine muscle-derived satellite cells (MDSCs) were added podocan protein and MDSCs were cultured in DM as control (Ctrl). After that cells were differentiated to 48 h or 72 h. **(A)** Immunofluorescence staining for supplementation of medium with podocan **(B)** Quantitative analysis of myotube fusion index of desmin-expressing cells as described in (A). **(C)** MYH3, and MyoG protein expression after podocan were added in bovine MDSCs differentiated for 48 and 72 h. **(D,E)** Quantification of podocan, MYH3, and MyoG protein expression from panel **(C)**. Statistical significance was calculated using the *t*-test; ^*^p < 0.05, ^∗∗^*p* < 0.01.

Next, we added purified podocan protein to the medium at a concentration of 1.0 mg/ml and allowed differentiation to proceed for 48 h and 72 h. Immunofluorescent staining showed that the number of myotubes increased in cultures with added podocan compared to that in cultures without added podocan protein (control) (Figure 4A). The fusion index increased by 21.7% (*p* < 0.01) in cells supplemented with podocan compared to that in control cells (Figure 4B). In addition, western blotting showed that MYH3 and MyoG increased upon addition of podocan (Figures 4C–E) and the raw dates showed in Supplementary Figure S7.

### Podocan Modulates Wnt/β-Catenin Signaling Pathway

**FIGURE 5:**
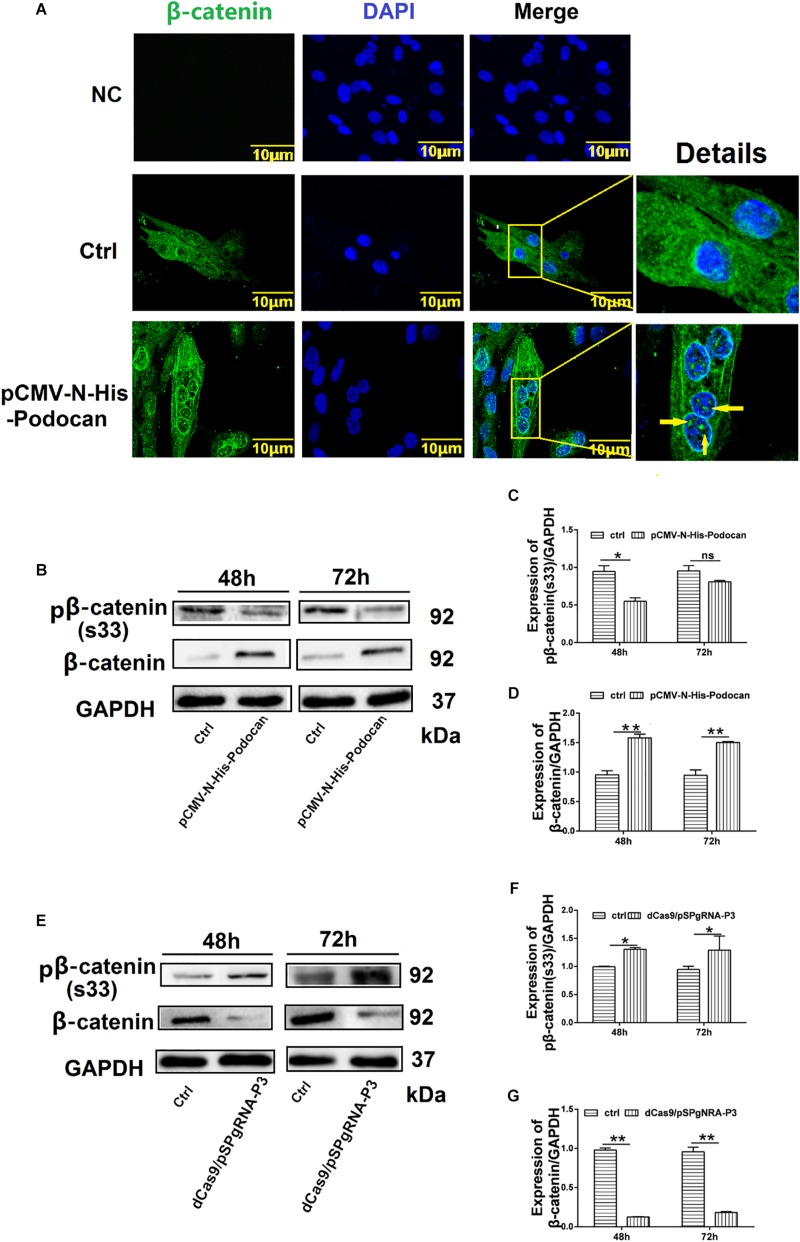
Podocan regulates the Wnt/β-catenin signaling pathway. **(A)** Bovine muscle-derived satellite cells (MDSCs) were transfected with pCMV-N-His as a control (Ctrl) or pCMV-N-His-Podocan. Immunofluorescence staining for β-catenin translocation after podocan overexpression in MDSCs. NC, negative control. Images were obtained with a Leica SP8 confocal microscope. Right, detailed localization of β-catenin. **(B)** β-catenin and phosphorylated β-catenin [pβ-catenin (S33)] protein expression with podocan overexpression. Bovine MDSCs were differentiated for 48 and 72 h. **(C,D)** Quantification of β-catenin and pβ-catenin(S33) protein expression from panel **(B)**. pβ-catenin(S33) protein expression was decreased, while β-catenin expression was increased after overexpressed podocan. **(E)** pβ-catenin (S33) and β-catenin expression after suppression of podocan. **(F,G)** Quantification of β-catenin and pβ-catenin (S33) protein expression from panel **(E)**. pβ-catenin(S33) protein expression was increased, while β-catenin expression was decreased after inhibit podocan.^*^p < 0.05, ^∗∗^*p* < 0.01; ns, no significant difference. Scale bar: 10 μm.

We next elucidated the mechanism by which podocan influences bovine MDSC differentiation by investigating the relationship between podocan and the Wnt/β-catenin signaling pathway. Immunostaining for β-catenin showed that overexpression of podocan led newly synthesized β-catenin to accumulate and translocate to the nucleus (Figure 5A). Importantly, western blotting showed that the expression of β-catenin protein increased, whereas phosphorylated β-catenin levels decreased upon podocan overexpression (Figures 5B–D). In contrast, β-catenin expression decreased and phosphorylated β-catenin levels increased significantly after podocan inhibition (*p* < 0.01; Figures 5E–G). Western blotting raw dates showed in Supplementary Figures S8, S9. These datas suggest that podocan regulates Wnt/β-catenin signaling in bovine MDSCs.

### Effect of Wnt/β-Catenin Signaling on Bovine MDSC Differentiation

**FIGURE 6:**
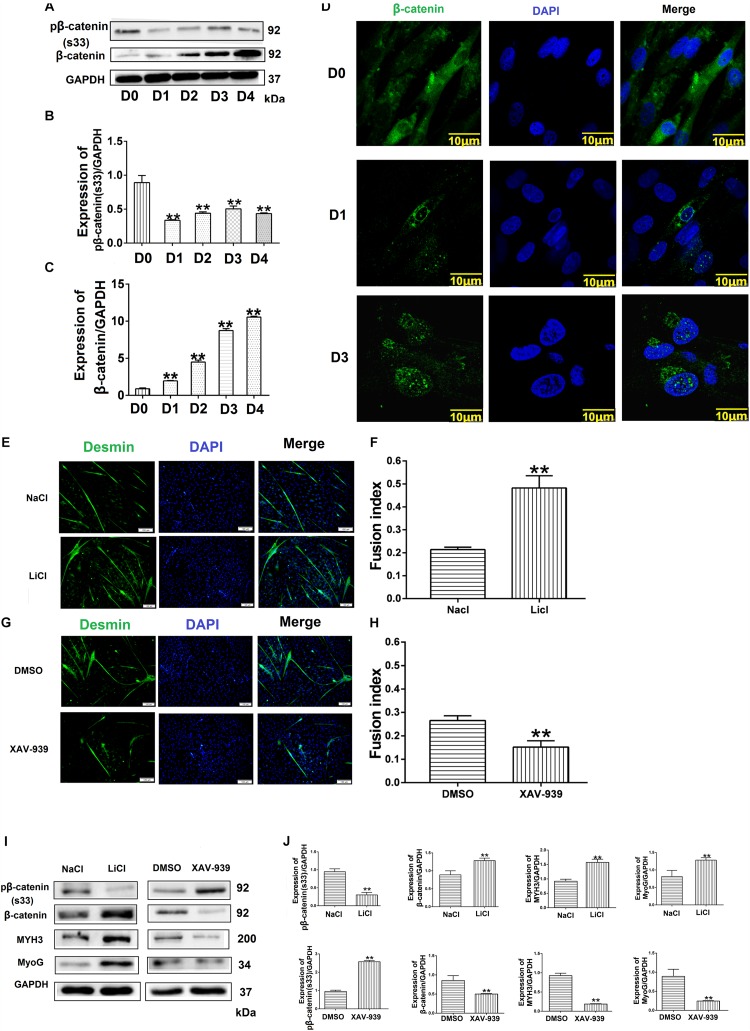
Wnt/β-catenin signaling promotes bovine muscle-derived satellite cell (MDSC) differentiation. **(A)** pβ-catenin (S33) and β-catenin protein expression at various days of differentiation. **(B,C)** Quantification of pβ-catenin (S33) and β-catenin protein expression from panel **(A)**. The results showed that pβ-catenin (S33) was decreased and β-catenin protein expression was increased in bovine MDSC differentiated into different days compared with D0. **(D)** Immunofluorescence staining to detect β-catenin translocation to the nucleus during proliferation and after induction of MDSC differentiation for 1 and 3 days. Scale bar: 10 μm **(E)** Immunofluorescence staining for the detection of desmin (green) after treating MDSCs with LiCl, followed by differentiation for 48 h. NaCl treatment was used as a control (Ctrl). Scale bar: 100 μm. **(F)** Quantitative analysis of myotube fusion index of desmin-expressing cells from panel **(E)**. MDSC Fusion index was increased after treated with LiCl compared with treated NaCl. **(G)** Immunofluorescence staining for the detection of desmin (green) after treating MDSCs with XAV-939, followed by differentiation for 48 h. DMSO treatment was performed as a control (Ctrl). Scale bar: 100 μm. **(H)** Quantitative analysis of myotube fusion index of desmin-expressing cells from (G). MDSC Fusion index was decreased after treated XAV-939 compared with treated DMSO. **(I)** pβ-catenin (S33), β-catenin, MYH3, and MyoG protein expression after treating MDSCs with LiCl and XAV-939, followed by differentiation for 48 h. **(J)** Quantification of pβ-catenin (S33), β-catenin, MYH3, and MyoG expression from panel **(I)**. The results showed that MYH3 and MyoG expression increased significantly after β-catenin expression increased compared with control, the opposite results when the expression level of β-catenin decreases. Statistical significance was calculated using the *t*-test and one-way ANOVA analysis; ^∗∗^*p* < 0.01.

To confirm the relationship between the Wnt/β-catenin pathway and MDSC differentiation, we investigated the effect of Wnt/β-catenin signaling on bovine MDSC differentiation. We measured the expression of phosphorylated β-catenin and β-catenin by western blotting and assessed the nuclear translocation of β-catenin by immunofluorescent staining at various stages of differentiation. The results showed that during differentiation, phosphorylated β-catenin levels decreased (Figures 6A,B). In addition, the expression of β-catenin increased (Figures 6A,C), as did its nuclear translocation, as differentiation progressed (Figure 6D). Western blotting raw dates showed in Supplementary Figure S10. Subsequently, we used LiCl and XAV-939 to activate and inhibit the Wnt/β-catenin pathway, respectively. Treatment with 10 mM LiCl, an inhibitor of glycogen synthase kinase (GSK3) that promotes β-catenin translocation to the nucleus, for 48 h extended the differentiation of bovine MDSCs. The myotube fusion index reached 48.23% for LiCl-treated cells compared to 21.38% for NaCl-treated control cells (*p* < 0.01; Figures 6E,F). XAV-939 selectively inhibits Wnt/β-catenin-mediated transcription by inhibiting tankyrase-1/2; upon 1 μM XAV-939 treatment, the fusion index was 15.19% compared to 28.85% for DMSO-treated controls (*p* < 0.01; Figures 6G,H). Moreover, western blotting showed that with LiCl treatment, the expression of β-catenin increased significantly (*p* < 0.01), whereas phosphorylated β-catenin levels decreased. We also found that MYH3 and MyoG levels were increased. In contrast, β-catenin expression decreased, whereas phosphorylated β-catenin levels increased. In contrast, upon XAV-939 treatment, MYH3 and MyoG protein expression decreased (*p* < 0.01) compared to that in DMSO-treated cells (Figures 6I,J). Western blotting raw dates showed in Supplementary Figure S11. Taken together, our results show that Wnt/β-catenin signaling influences bovine MDSC differentiation.

### Podocan Affects MDSC Differentiation Through the Wnt/β-Catenin Signaling Pathway

**FIGURE 7:**
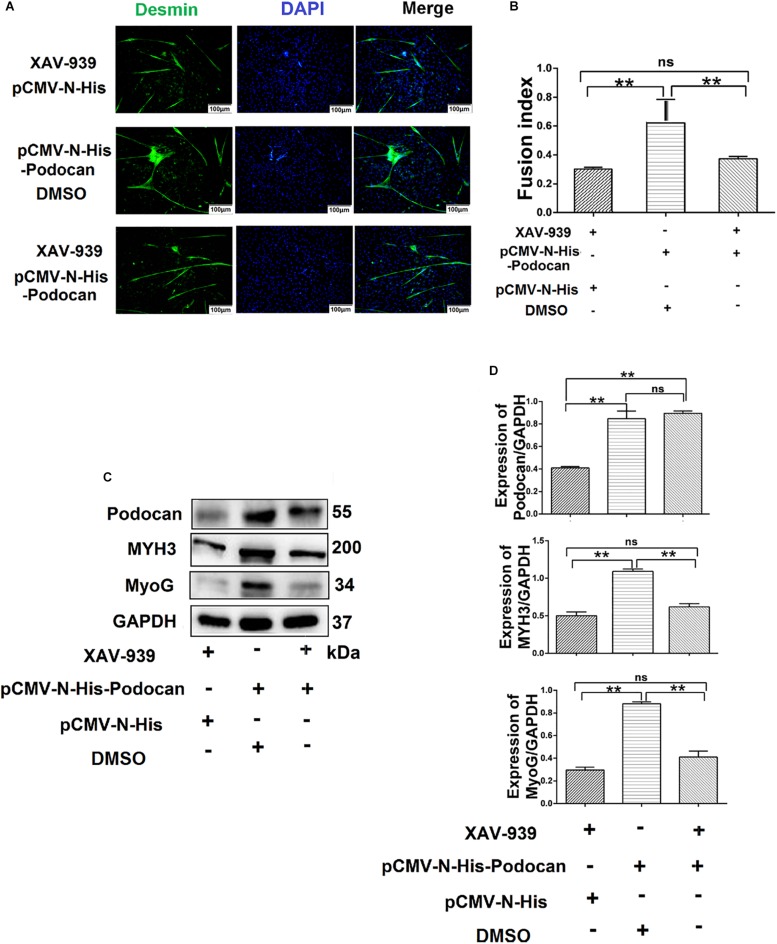
Podocan influences bovine muscle-derived satellite cell (MDSC) differentiation via the Wnt/β-catenin signaling pathway. **(A)** Immunofluorescence staining to determine the effects of podocan overexpression on cell differentiation after inhibiting the Wnt signaling pathway. The first group of cells was transfected with pCMV-N-His and treated with XAV-939 for 48 h; the second group was transfected with pCMV-N-His-Podocan and treated with DMSO for 48 h; the third group was transfected with pCMV-N-His-Podocan and treated with XAV-939 for 48 h. Scale bar: 100 μm. **(B)** Quantitative analysis of myotube fusion index of desmin-expressing cells as described in panel **(A)**. Fusion index was higher in second group than in first and third groups. **(C)** Podocan, MYH3, and MyoG protein expression after treatment with XAV-939, overexpression of podocan, or both. **(D)** Quantification of podocan, MYH3, and MyoG protein expression from panel **(C)**. The level of podocan protein expression in second and third group were no significant difference. MYH3 and MyoG proteins expression in second group was higher than that in third group. Statistical significance was calculated using the one-way ANOVA analysis; ^∗∗^*p* < 0.01; ns, no significant difference.

Based on the above results, whether podocan still exerts an effect on cell differentiation when the Wnt/β-catenin signaling pathway is inhibited remained uncertain. Therefore, we evaluated the differentiation of MDSCs upon podocan overexpression and XAV-939 treatment to inhibit the Wnt/catenin signaling pathway. After differentiation of cells for 48 h, the myotube fusion index was not significantly different between MDSCs overexpressing podocan and treated with XAV-939 and MDSCs transfected with an empty vector and treated with XAV-939 (*p* > 0.05). Moreover, the myotube fusion index of MDSCs overexpressing podocan and treated with XAV-939 was significantly lower than that of MDSCs overexpressing podocan and treated with DMSO (Figures 7A,B). MYH3 and MyoG protein expression levels were also significantly downregulated by XAV-939 treatment and transfection with pCMV-N-His-Podocan, as compared with those in MDSCs overexpressing podocan and treated with DMSO (*p* < 0.01; Figures 7C,D). Western blotting raw dates showed in Supplementary Figure S12. Therefore, the function of podocan in promoting the differentiation of bovine MDSCs is suppressed when Wnt/β-catenin signaling is inhibited.

### Podocan Interacts With Wnt4

**FIGURE 8:**
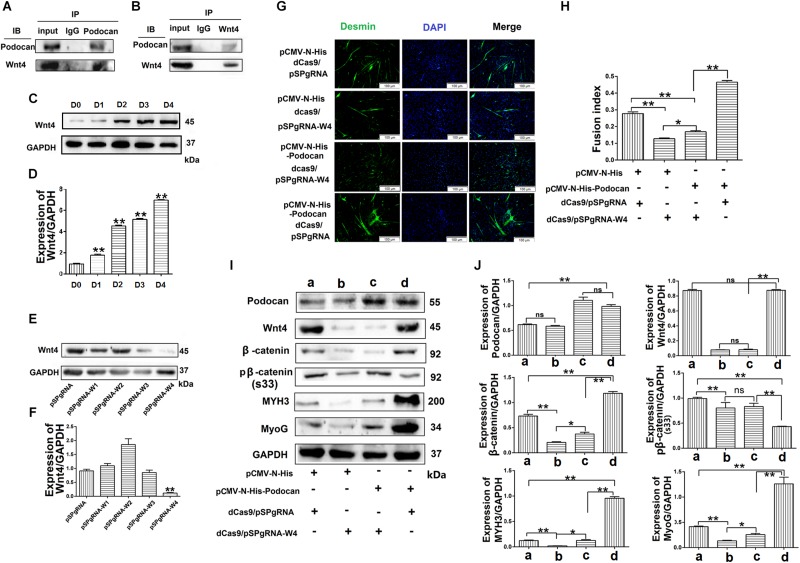
Podocan promotes bovine muscle-derived satellite cell (MDSC) differentiation by regulating Wnt4-β-catenin. **(A)** Podocan overexpression in cells after transfection and differentiation for 72 h. Podocan antibodies were used for immunoprecipitation, and Wnt4 was detected by western blotting. **(B)** Wnt4 antibodies were used for immunoprecipitation, and podocan was detected by western blotting. **(C)** The expression of Wnt4 during MDSC differentiation. **(D)** Quantification of Wnt4 protein expression from panel **(C)**. **(E)** Wnt4 protein expression after co-transfection with dCas9 and pSPgRNA-W(1–4) plasmids. **(F)** Quantification of Wnt4 protein expression from panel **(E)** Western blotting results showed that Wnt4 protein expression was lowest in co-transfected with dCas9 and pSPgRNA-W4 group. **(G)** Immunofluorescence was performed to detect the effects of podocan overexpression on cell differentiation after inhibiting Wnt4 expression. The first group was transfected with pCMV-N-His, dCas9, and pSPgRNA. The second group was transfected with pCMV-N-His, dCas9, and pSPgRNA-W4. The third group was transfected with pCMV-N-His-Podocan, dCas9, and pSPgRNA-W4. The last group was transfected with pCMV-N-His-Podocan, dCas9, and pSPgRNA. Scale bar: 100 μm. **(H)** Quantitative analysis of myotube fusion index of desmin-expressing cells as described in panel **(G)**. Fusion index in third group was lower than in last group significantly. **(I)** Podocan, Wnt4, β-catenin, pβ-catenin (S33), MYH3, and MyoG protein expression. **(J)** Quantification of podocan, Wnt4, β-catenin, pβ-catenin (S33), MYH3, and MyoG protein expression from panel **(I)**. The results showed that podocan was overexpressed in c and d groups; Wnt4 was inhibited in b and c groups; β-catenin protein expression in c group were lower than that in d group, MYH3 and MyoG protein expression in c group lower than in d group. Statistical significance was calculated using the one-way ANOVA analysis; ^*^p < 0.05, ^∗∗^*p* < 0.01; ns, no significant difference.

To further determine the mechanism through which podocan regulates the Wnt pathway, we identified podocan interactors by co-immunoprecipitation and sequencing of the resulting gel. The results are shown in Table 3. Based on these results, we identified Wnt4, which is a Wnt family protein, as a potential binding partner of podocan. We then further examined the interaction between podocan and Wnt4. Podocan was overexpressed in bovine MDSCs, and lysates were first immunoprecipitated with anti-podocan antibodies and then blotted using anti-Wnt4 antibodies. The raw dates showed in Supplementary Figure S13 and the results showed that Wnt4 was present in the protein complexes immunoprecipitated using anti-podocan antibodies (Figure 8A). Moreover, when lysates were immunoprecipitated with anti-Wnt4 antibodies and blotted using anti-podocan antibodies, the results confirmed the association between podocan and Wnt4 (Figure 8B).

**TABLE 3:** Analysis and identification of protein species that bind to podocan.

**Entry**	**Gene symbol**	**Protein name**	**Unique peptide count**	**Coverage percent**
1	Myh9	Myosin, heavy polypeptide 9	15	6.36%
2	TPM1	Tropomyosin alpha-1 chain	15	41.9%
3	ACTG1	Actin, cytoplasmic 2	12	23.73%
4	GAPDH	Glyceraldehyde-3-phosphate dehydrogenase	9	24.32%
5	Myh10	Myosin-10	8	3.8%
6	ACTR2	Actin-related protein 2	5	6.35%
7	ANXA5	Annexin A5	5	15.26%
8	WIPS1	WNT1 inducible signaling pathway protein 1	2	1.67%
9	HSPA8	Heat shock cognate 71	7	8.46%
10	PRPH	Peripherin	2	3.26%
11	CD9	CD9 antigen	2	6.64%
12	ANXA1	Annexin A1	2	5.49%
13	MYL6	Myosin light polypeptide 6	2	9.27%
14	GLYCAM1	Glycosylation-dependent cell adhesion molecule 1	2	8.50%
15	MYO1B	MYO1B	2	0.88%
16	HIST1H4H	Histone H4	2	21.36
17	WNT4	Protein Wnt	1	2.46%
18	HIST1H1A	Histone H1.1	1	5.05%
19	INSL3	Insulin-like 3	1	4.55%
20	TUBB2B	Tubulin beta-2B chain	1	2.02%

### Podocan Regulates Wnt4/β-Catenin Signaling to Promote Bovine MDSC Differentiation

To test whether podocan functions through Wnt4, we overexpressed podocan after Wnt4 inhibition and measured changes in β-catenin levels and cell differentiation. First, we detected the expression of Wnt4 during MDSC differentiation. The result showed that the expression of Wnt4 increased after MDSC differentiation (Figures 8C,D). The raw dates showed in Supplementary Figure S14. Next, we used western blotting to screen for vectors that inhibit *Wnt4* gene expression. The results showed that pSPgRNA-W4 was optimal (Figures 8E,F and Supplementary Figure S15), and it was therefore used for all subsequent Wnt4 knockdown experiments. Thereafter, immunofluorescence results showed decreased myotube fusion upon Wnt4 inhibition. MDSCs with knockdown of Wnt4 combined with podocan overexpression had significantly lower myotube fusion rates than those with podocan overexpression only (*p* < 0.01; Figures 8G,H). Western blotting results showed that MYH3 and MyoG protein expression decreased after Wnt4 knockdown. Moreover, β-catenin, MYH3, and MyoG levels were lower in cells with Wnt4 knockdown combined with podocan overexpression than in cells with only podocan overexpression. However, MYH3 and MyoG protein expression levels were lower with Wnt4 knockdown only than with Wnt4 knockdown combined with podocan overexpression (Figures 8I,J and Supplementary Figure S16).

## Discussion

Podocan is the fifth-identified member of the SLRP family. The members of the SLRP family have been studied owing to their multiple functions in various cell types and tissues (Mochida et al., 2011). However, the expression and function of podocan in bovine MDSC differentiation has not been previously reported. The results of our previous deep sequencing study demonstrated that the expression of podocan mRNA in bovine MDSCs on D3 of differentiation was 11.2 times higher than that in undifferentiated (D0) cells (Tong et al., 2015). The mRNA expression levels of differentially expressed genes from this analysis are shown in Table 4. Moreover, we have reported that Podocan affects the differentiation of C2C12, but the molecular mechanism by which Podocan works is not clear (Liu et al., 2019) Therefore, we speculated that podocan may play an important role in this process. First, our research showed that the expression of podocan is upregulated during the differentiation of bovine MDSCs, which is consistent with our deep sequencing results (Tong et al., 2015). Thus, identifying the genes that play essential roles in muscle differentiation is crucial. However, the associated molecular mechanism of action remained unclear.

**TABLE 4:** Differentially expressed genes between differentiated MDSCs (D3) and undifferentiated MDSCs according to deep sequencing analysis.

**Gene symbol**	**log2Ratio (Fold Change)**	**Gene symbol**	**log2Ratio (Fold Change)**
MYBPC1	11.9	OBSCN	11.5
ABRA	11.3	Podocan	11.2
DHRS3	11.1	SLC9A3R2	10.7
EGR1	10.5	ECM2	10.4
TCEA3	10.2	TCP11L2	10.1

The process of cell differentiation is regulated by multiple signaling pathways (Xie et al., 2018). Extracellular signals are very important for these pathways, and they include soluble factors, cell–cell interaction factors, and ECM proteins (Roskelley et al., 1995; Gumbiner, 1996; Daley et al., 2008). ECM proteins are highly effective and selective modulators of migration, proliferation, and differentiation (ten Dijke and Arthur, 2007; Daley et al., 2008; Liu et al., 2017). There is a substantial number of extracellular proteins that act as extracellular signaling coordinators (Dellett et al., 2012). One of the components of the ECM is a group of non-collagenous glycoproteins known collectively as the SLR protein family (Shimizu-Hirota et al., 2004b). SLRPs have a common leucine-rich repeat domain and a general consensus sequence (Hocking et al., 1998). Previous studies have demonstrated that multiple signaling pathways are evoked by SLRPs (Schaefer and Iozzo, 2008). For example, biglycan and decorin, which comprise the first canonical class of SLRPs, are dependent on transforming growth factor-β signaling in the palatal shelves prior to adhesion (Schaefer and Iozzo, 2008; Ibrahim et al., 2017). The integrin/FAK signaling pathway is required for adhesion to the ECM and controls the proliferation and differentiation of myoblasts (Han et al., 2011). However, the signaling pathway through which podocan acts as an ECM protein to promote the differentiation of bovine MDSCs was previously unknown. Based on previous research showing upregulation of the Wnt-TCF pathway in SMCs from podocan-deficient mice, we speculated that the effect of podocan on the differentiation of bovine MDSCs may involve the Wnt pathway (Hutter et al., 2013).

The key step in the Wnt/β-catenin signaling pathway is the regulation of the stability of the Wnt effector β-catenin (Schaefer et al., 2018). Studies have shown that β-catenin is strongly upregulated at the onset of differentiation and undergoes nuclear translocation as differentiation progresses in human primary CD56Pos satellite cell-derived cells (Agley et al., 2017). Free β-catenin is localized in the cytoplasm. When the Wnt signaling pathway is activated, β-catenin is translated into the nucleus or degraded upon phosphorylation (Fagotto, 2013). Herein, we thus confirmed an association between podocan and the Wnt/β-catenin pathway.

To date, conflicting reports have proposed distinct roles for the canonical Wnt/β-catenin pathway during muscle differentiation and proliferation. Wnt signaling plays key roles in cell fate decision and stem cell homeostasis during normal development. The suppression of Wnt signaling factors may balance the proliferation and differentiation of myogenic cells (Alexander et al., 2013). MyoG mediates the canonical Wnt/β-catenin-dependent activation of follistatin and the induction of myogenic differentiation (Jones et al., 2015). An earlier work showed that Wnt signaling regulates myogenic differentiation in the developing avian wing (Anakwe et al., 2003). The Wnt1/β-catenin pathway acts in muscle cell differentiation through the activation and recruitment of MuSC-like reserve myoblasts for fusion with myotubes *in vitro* (Rochat et al., 2004). In contrast, β-catenin signaling in proliferating satellite cells directs these cells toward the self-renewal pathway (Perez-Ruiz et al., 2011). Canonical Wnt signaling induces satellite-cell proliferation during adult skeletal muscle regeneration (Otto et al., 2008). Intriguingly, *in vivo* experiments have suggested that canonical Wnt signaling is not required for muscle regeneration or satellite cell self-renewal (Murphy et al., 2014). However, the roles of Wnt/β-catenin in the differentiation of bovine MDSCs was previously unknown.

Next, we used LiCl to activate the Wnt signaling pathway, based on a previous study (Klein and Melton, 1996). In addition, XAV-939 was used to inhibit Wnt signaling (Huang et al., 2009). However, these two compounds had not previously been used on bovine MDSCs. Therefore, we first tested the efficiency of the two drugs in activating or inhibiting the Wnt signaling pathway using western blotting. Our results demonstrate that Wnt/β-catenin signaling promotes bovine MDSC differentiation. Furthermore, podocan-mediated bovine MDSC differentiation was blocked when the Wnt/β-catenin pathway was inhibited (Figure 7).

Wnt signaling is a critical regulator of embryonic development, tissue regeneration, and homeostasis. Various Wnt ligands can elicit different responses depending on the receptors and cell context (Janda et al., 2017). Wnts are also among the most influential group of signals supporting stem cells within their niches, regulating self-renewal or differentiation status depending on context (Krausova and Korinek, 2014). Furthermore, Wnt signaling is a key pathway involved in limb regeneration and appears to be regulated during numerous processes, including cell differentiation and muscle growth, with Wnt4 being a key mediator (Liu et al., 2018). Previous studies showed that Wnt4 is essential for normal mammalian lung development (Caprioli et al., 2015). Wnt4 also activates the canonical β-catenin pathway and negatively regulates myostatin during C2C12 myogenesis (Bernardi et al., 2010). Co-immunoprecipitation is one of the most widely used methods for identifying novel proteins associated with a protein of interest or for determining complex formation between known proteins (Takahashi, 2015). Our co-immunoprecipitation results showed that podocan directly interacts with Wnt4 and that the effect of podocan on MDSC differentiation was blocked upon inhibition of Wnt4. We speculate that this may be due to the structure of podocan. As an extracellular glycoprotein, most members of the SLRP protein family covalently bind to glycosaminoglycans (GAG) to form proteoglycan (PG), which can activate various cell surface receptors, growth factors, cytokines, and other ECM components to affect cell function and activate Wnt/β-catenin signaling pathways (Dellett et al., 2012). Further studies are needed to determine whether the glycosyl (or GAG group) in the podocan molecule has an effect on the binding of podocin and activation of Wnt4. Taken together, our results show that podocan regulates the Wnt/β-catenin signaling pathway by interacting with Wnt4.

This study demonstrated that podocan promotes bovine muscle satellite cell differentiation and elucidated its underlying mechanism. In particular, podocan promotes the upstream activation of the Wnt/β-catenin signaling pathway. However, whether the mechanism of podocan in regulating the differentiation of MDSCs is applicable to other species requires further study. In addition, podocan may interact with other molecular targets and regulate other signaling pathways, warranting further investigation.

## Conclusion

In conclusion, podocan promotes bovine MDSC differentiation by regulating the Wnt4/β-catenin signaling pathway, providing the basis for future research and the improvement of beef quality.

## Data Availability

The datasets used and/or analyzed during the current study are available from the corresponding author on reasonable request.

## Author Contributions

SL, DL, and YY conceived and designed the experiments. SL and YF performed experiments. ZY and HT performed the data analysis. SL wrote the manuscript, checked the results and revised the manuscript. All authors read and approved the final manuscript.

## Conflict of Interest Statement

The authors declare that the research was conducted in the absence of any commercial or financial relationships that could be construed as a potential conflict of interest.
