# Supplementary material for: Podocan Promotes Differentiation of Bovine Skeletal Muscle Satellite Cells by Regulating the Wnt4-β-Catenin Signaling Pathway
Source: Front Physiol. 2019 Aug 7;10:1010. doi: 10.3389/fphys.2019.01010 (PMC6692459; doi:10.3389/fphys.2019.01010)
Supplement: Supplementary file 1 [file Data_Sheet_1.doc]

**Supplementary materials**

Supplementary figure 1


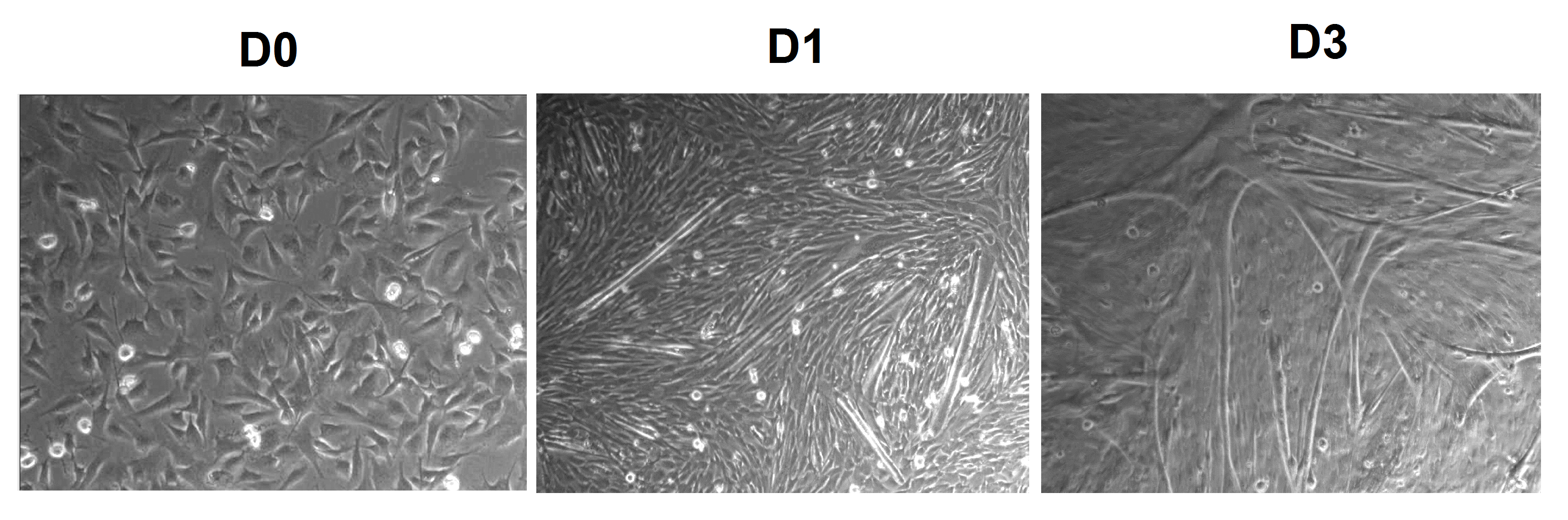


Figure S1 The phase contrast pictures of the cells which were started from undifferentiated to differentiated

Supplementary figure 2


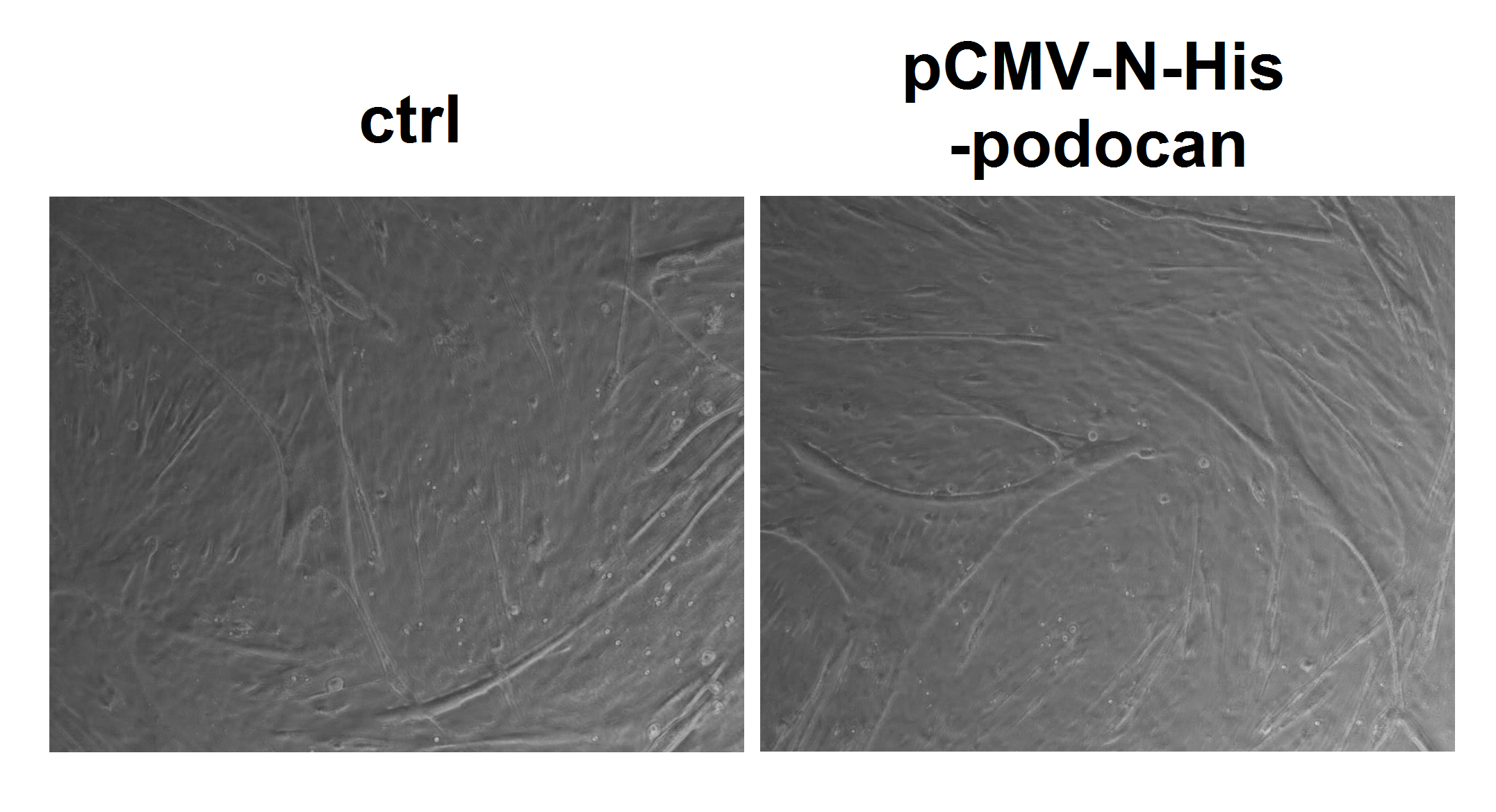


Figure S2 The phase contrast pictures of the differentiating cells after overexpression of podocan

Supplementary figure 3


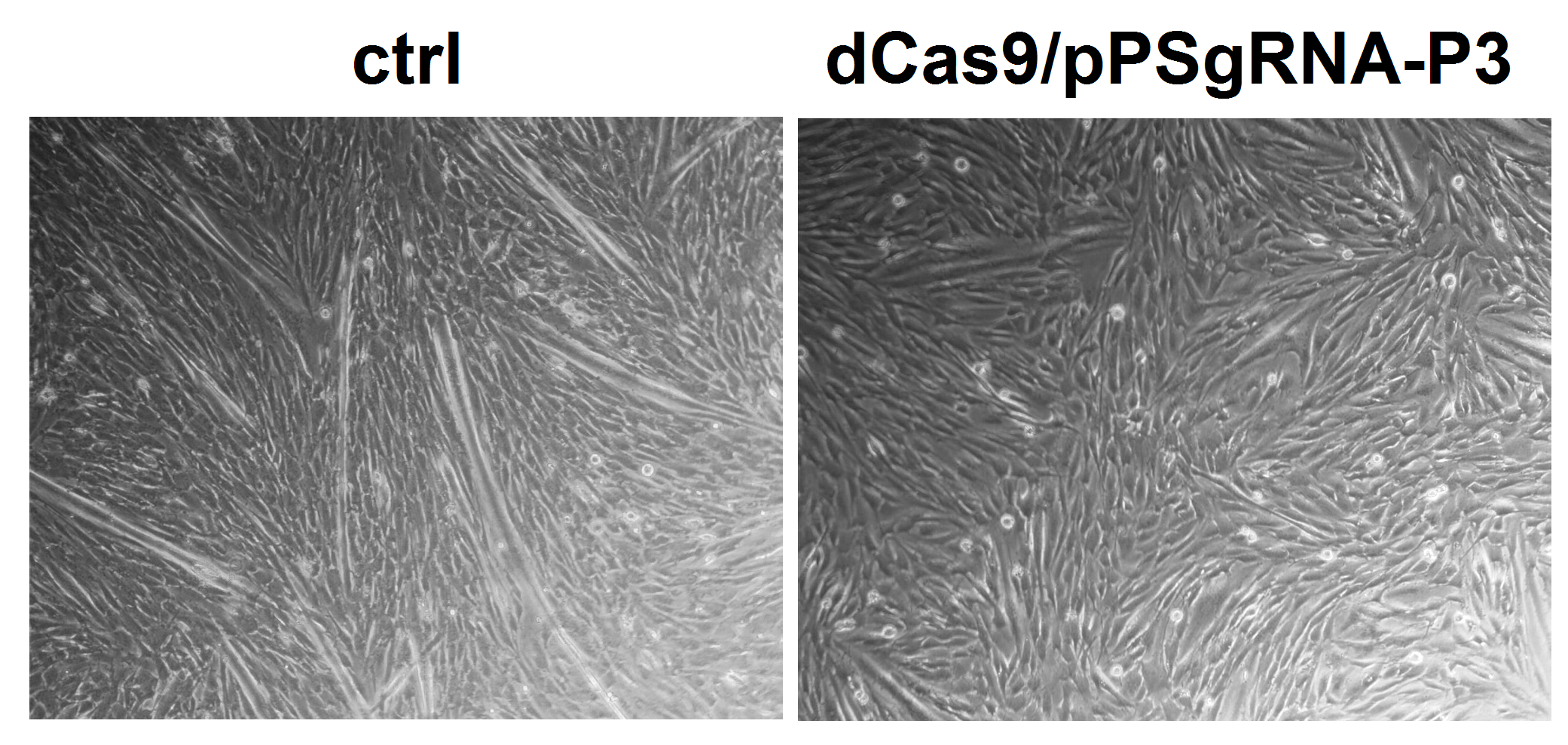


Figure S3 The phase contrast pictures of the differentiating cells after knockdown podocan

Supplementary figure 4


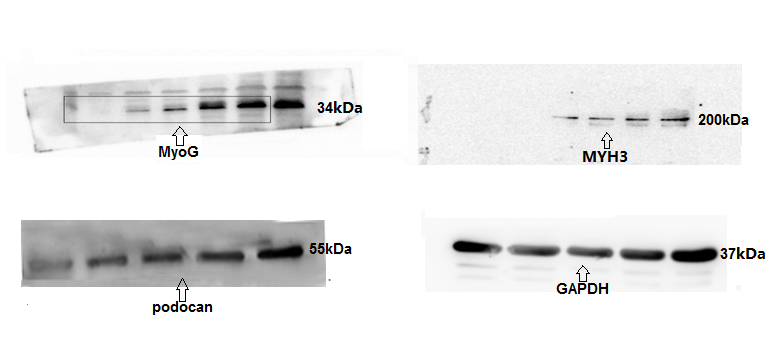


Figure S4 Western blotting raw dates of Figure 1A

Supplementary figure 5


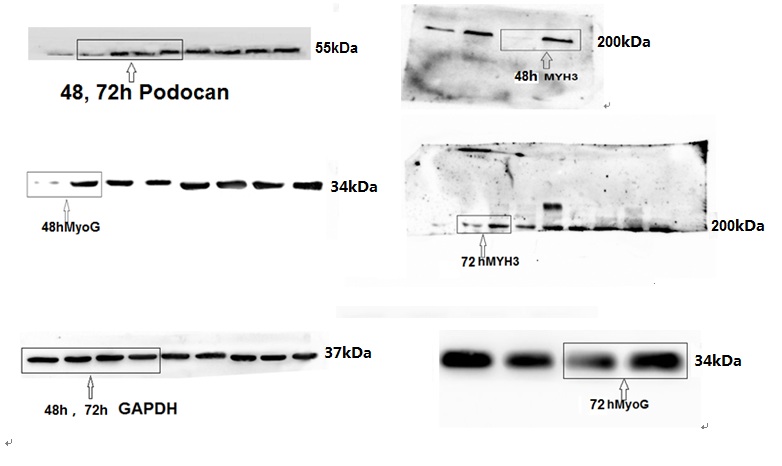


Figure S5 Western blotting raw dates of Figure 2F

Supplementary figure 6


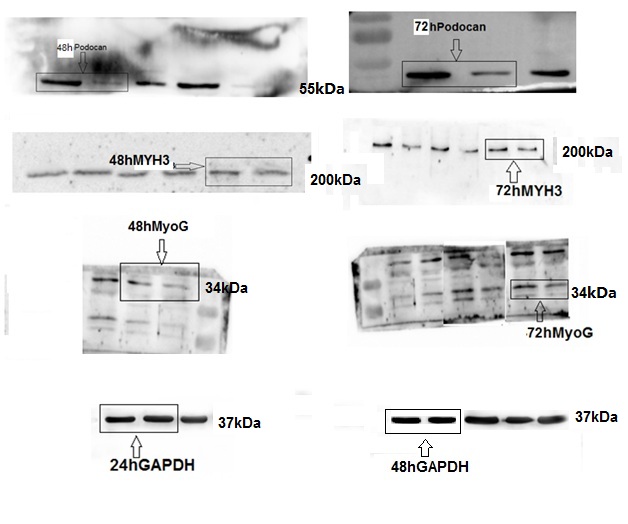


Figure S6 Western blotting raw dates of Figure 3I

Supplementary figure 7


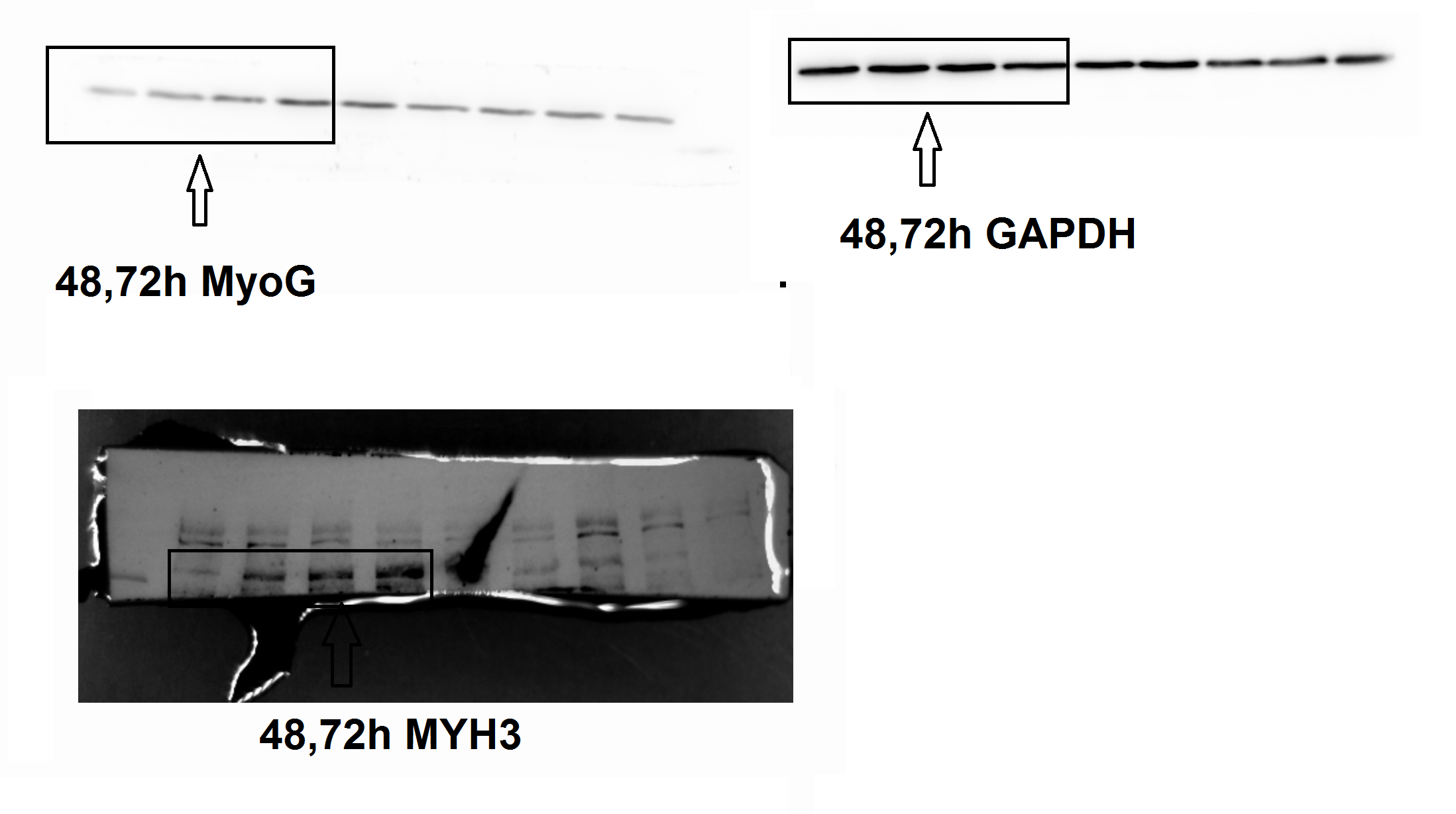


Figure S7 Western blotting raw dates of Figure 4C

Supplementary figure 8


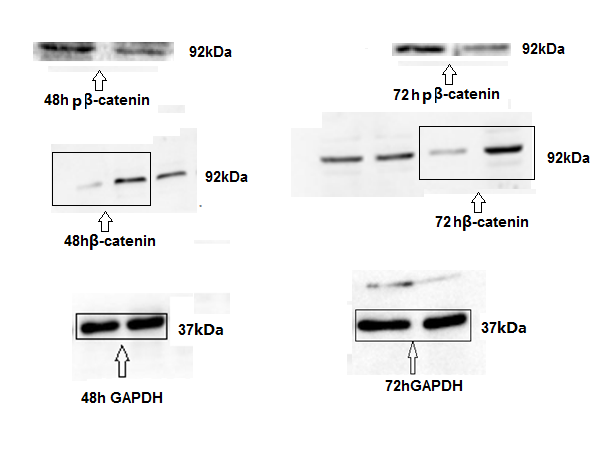


Figure S8 Western blotting raw dates of Figure 5B

Supplementary figure 9


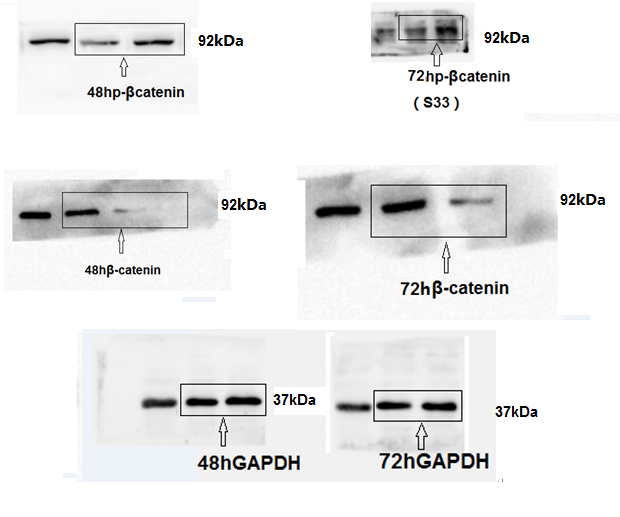


Figure S9 Western blotting raw dates of Figure 5E

Supplementary figure 10


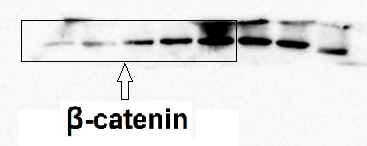

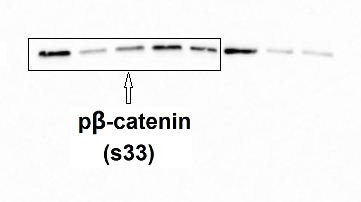


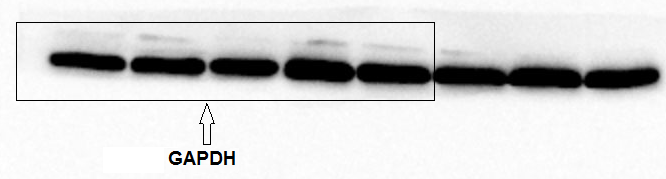


Figure S10 Western blotting raw dates of Figure 6A

Supplementary figure 11


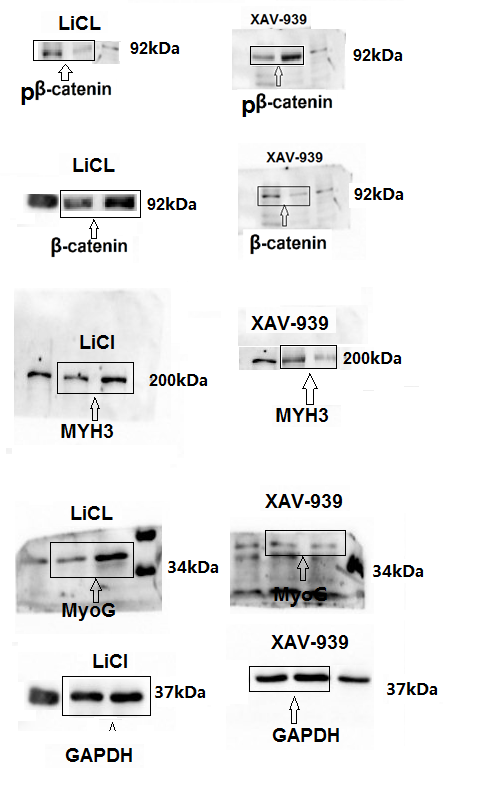


Figure S11 Western blotting raw dates of Figure 6I

Supplementary figure 12


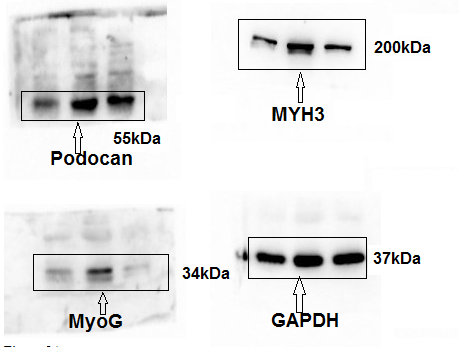


Figure S12 Western blotting raw dates of Figure 7C

Supplementary figure 13


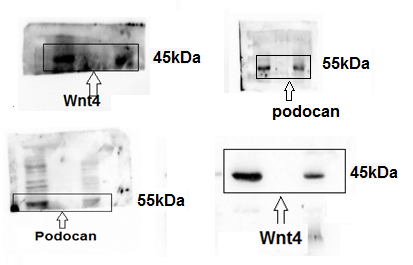


Figure S13 Western blotting raw dates of Figure 8A and 8B

Supplementary figure 14


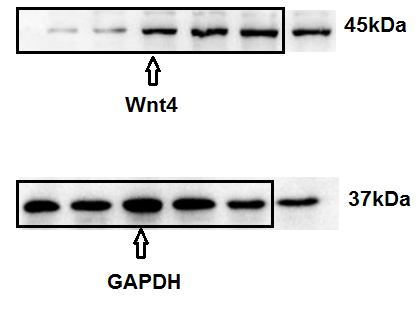


Figure S14 Western blotting raw dates of Figure 8C

Supplementary figure 15


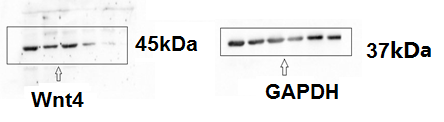


Figure S15 Western blotting raw dates of Figure 8E

Supplementary figure 16


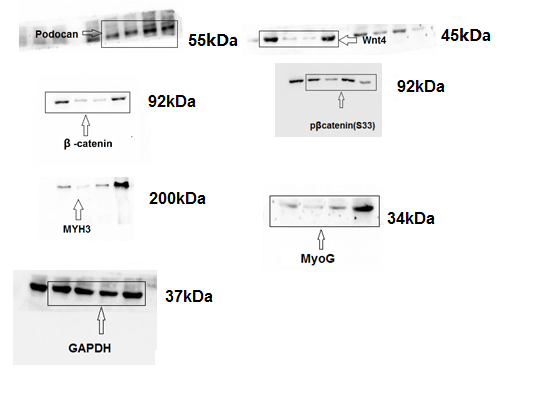


Figure S16 Western blotting raw dates of Figure 8I
